# Optimising Health Literacy and Access of Service Provision to Community Dwelling Older People with Diabetes Receiving Home Nursing Support

**DOI:** 10.1155/2016/2483263

**Published:** 2016-09-07

**Authors:** Dianne Goeman, Sue Conway, Ralph Norman, Jo Morley, Rona Weerasuriya, Richard H. Osborne, Alison Beauchamp

**Affiliations:** ^1^RDNS Institute, St Kilda, VIC, Australia; ^2^Central Clinical School, Monash University, Melbourne, VIC, Australia; ^3^RDNS, South Site, Bentleigh, VIC, Australia; ^4^RDNS, East Site, Knox, VIC, Australia; ^5^RDNS, West Site, Ravenhall, VIC, Australia; ^6^Health Systems Improvement Unit, Deakin University Centre for Population Health Research, School of Health and Social Development, Geelong, VIC, Australia

## Abstract

*Background*. Health literacy is the ability to access, understand, and use information and services for good health. Among people with chronic conditions, health literacy requirements for effective self-management are high. The Optimising Health Literacy and Access (Ophelia) study engaged diverse organisations in the codesign of interventions involving the Health Literacy Questionnaire (HLQ) needs assessment, followed by development and evaluation of interventions addressing identified needs. This study reports the process and outcomes of one of the nine organisations, the Royal District Nursing Service (RDNS).* Methods*. Participants were home nursing clients with diabetes. The intervention included tailored diabetes self-management education according to preferred learning style, a standardised diabetes education tool, resources, and teach-back method.* Results*. Needs analysis of 113 quota-sampled clients showed difficulties managing health and finding and appraising health information. The service-wide diabetes education intervention was applied to 24 clients. The intervention was well received by clients and nurses. Positive impacts on clients' diabetes knowledge and behaviour were seen and nurses reported clear benefits to their practice.* Conclusion*. A structured method that supports healthcare services to codesign interventions that respond to the health literacy needs of their clients can lead to evidence-informed, sustainable practice changes that support clients to better understand effective diabetes self-management.

## 1. Introduction

Health literacy has been described as “the cognitive and social skills which determine the motivation and ability of individuals to gain access to, understand and use information in ways which promote and maintain good health” [1]. Health literacy goes beyond the individual, however, as the skills, preferences, and expectations of healthcare providers (doctors, nurses, and home health workers) also play a critical role in creating environments that enable people with low health literacy to get access and to use services equitably [2]. It is therefore essential that health professionals and healthcare services are active in identifying and responding to the needs of their clients. This is particularly relevant for people with chronic conditions such as diabetes in whom the health literacy requirements for effective self-management are high [3]. Given the increasing prevalence of diabetes and other chronic conditions in the community [4, 5], new approaches that focus on health literacy as an integrated component of care are important to consider. One such approach is the Optimising Health Literacy and Access (Ophelia) process, a structured method that supports healthcare services and providers to codesign interventions that respond to the health literacy needs of their clients [6]. An underlying principle of the Ophelia approach is that interventions are locally relevant. This is important because health literacy is context-specific [7], and interventions that are developed in one population or setting may not be relevant in other settings. Approaches such as Ophelia can be used on a small scale to codesign interventions that are appropriate for and specific to the needs of particular client groups or communities.

The Ophelia process was developed and tested in a proof-of-concept study across nine diverse healthcare services in Victoria, Australia (hereafter referred to as Ophelia Victoria) [6, 8]. This paper describes the process as undertaken in one healthcare service, RDNS, a large home nursing service provider delivering care across metropolitan Melbourne [8]. On commencement of the project, each participating healthcare service was asked to identify a priority group in whom health literacy was thought to be a contributing factor to incomplete access to services or poorer health outcomes. The home nursing service identified that many clients with diabetes struggled with independent self-management of their condition and that education of these clients was not consistent throughout the service.

The overall aim of the project was therefore to improve the service's approach to diabetes education so that clients were better supported to self-manage their condition. In line with the Ophelia process, the subaims were to (i) conduct an assessment of health literacy among clients with diabetes, (ii) develop an intervention to address any identified needs, and (iii) evaluate the outcome of the intervention. This paper reports on the Ophelia process as undertaken within the service, including clients' health literacy-related outcomes and the perceptions of staff about barriers to delivery of the intervention and any impact upon their clinical practice or client outcomes.

## 2. Methods

### 2.1. Study Design

A three-phase codesign study was used to achieve the aims of the study. Phase one, a needs assessment, involved undertaking a health literacy survey in a cohort of community-based clients with diabetes who were receiving home nursing services [8]. Clinicians from the service then generated a range of potential intervention ideas in response to the issues identified by the survey. Phase two of the study involved selection of a set of intervention ideas considered to be most likely to achieve the study aim, then combining these ideas to form one overall intervention which was further refined through small quality improvement cycles. In Phase three, the selected intervention was implemented and evaluated more broadly within the service, measured by client outcomes and staff experiences.

### 2.2. Setting

Seven RDNS sites across the Melbourne metropolitan area participated in a health literacy survey of their clients and implementation of the intervention. Health literacy data were collected for the period July 2013 to December 2014 and the intervention was implemented and evaluated between September 2014 and February 2015.

#### 2.2.1. Phase One: Needs Assessment

*Participants for Phase One*. All home nursing clients with diabetes, from the seven study sites, were considered suitable for participation on the basis of belonging to the priority group identified at study commencement. Inclusion criteria were being aged 18 years or over, cognitively able to participate, and able to provide informed consent. Participants and data collection are described in detail elsewhere [8].

*Data Collection for Phase One*. Eligible clients were approached by their attending generalist nurse to undertake the survey. To maximise the participation rate of people with low health literacy, consenting clients were invited to either complete the Health Literacy Questionnaire (HLQ) by themselves or to have assistance from family members, carers, or nursing staff. The HLQ is a widely used and well-validated 44-item measure that captures the concept of health literacy across nine distinct domains [8]. The nine scales of the HLQ can be used as a needs diagnostic tool and an outcomes measure. Importantly, the scales allow for the development of health literacy “profiles” describing an individual's health literacy needs and strengths [9]. The nine HLQ scales are (1) feeling understood and supported by healthcare providers; (2) having sufficient information to manage my health; (3) actively managing my health; (4) social support for health; (5) appraisal of health information; (6) ability to actively engage with healthcare providers; (7) navigating the healthcare system; (8) ability to find good health information; and (9) understanding health information enough to know what to do. In combination, these scales provide a profile of a person's health literacy strengths and limitations. Data were also collected on demographic and health status [8].

*Data Analysis for Phase One*. As described elsewhere [6], cluster analysis of the HLQ alongside demographic data was then undertaken using SPSS [10]. This statistical technique allows identification of groups of clients placed into clusters on the basis of having similar health literacy profiles across the nine HLQ dimensions. The pattern of health literacy scores within each cluster then informs the development of short narratives (vignettes). These vignettes describe an archetypal individual with a specific health literacy profile of strengths and weaknesses. Each vignette details how that person's health literacy profile might impact upon their ability to manage their health and interact with the services around them. Demographic data were analysed using STATA [11].

#### 2.2.2. Phase Two: Cocreation of the Intervention

In a workshop setting, highly experienced Clinical Diabetes Educators and a Senior Research Fellow from the home nursing service discussed the clinical vignettes and developed potential intervention ideas in response to the health literacy needs identified within. Following the workshop, a set of these intervention ideas was selected as being likely to meet the aims of the study. Program Logic models [12] were developed to describe how the intervention ideas could lead to the desired outcome, with selection of the final set of interventions based on further consensus meetings, including a cross-site meeting with the other eight organisations participating in the Ophelia study from across Victoria [6] in which project teams shared their intervention ideas and provided peer feedback to each other. Following this cross-site meeting, a single site from the home nursing service undertook pilot testing and refinement of their intervention processes and materials using Plan, Do, Study, Act (PDSA) cycles.

#### 2.2.3. Phase Three: Implementation and Evaluation

From Phase two, the final selected intervention set included three components (described in more detail in the results section):

1. Use of guidelines and checklist for education of clients with diabetes.
2. The services' generalist nurses trained in using the teach-back method of patient education.
3. Development of an online library of resource material for generalist nurses to use when providing education to clients with diabetes.

Phase three involved broader dissemination of the intervention within seven sites of the home nursing service as follows.

*Participants for Phase Three*

*Clients*. Over a five-month period, across the seven home nursing sites from Phase one (including from the site used for pilot testing), convenience sampling was used to identify all eligible clients with diabetes who required education for self-management of their diabetes. Exclusion criteria included being cognitively impaired and having difficulty understanding and retaining information (likely to be the clients who were not routinely provided with detailed education but where others manage most of the care for the client). Clients not speaking or reading English were also excluded. Of note, the intervention was delivered as “usual care” by participating nurses to all clients receiving diabetes education. Only those clients who consented to be involved in evaluation were included as study participants for this phase.

*Nurses*. All generalist nurses at the seven participating sites were invited to a training session and introduced to the use of teach-back and the diabetes education guidelines and checklist. These sessions were facilitated by the Clinical Diabetes Educators who had been involved in the study from the start.

*Data Collection for Phase Three*. Data collection activities were undertaken by generalist nurses from the seven home nursing sites, with this phase of the project managed by the Clinical Diabetes Educators. Generalist nurses who had attended the training sessions were asked to invite eligible clients to participate in evaluation of the intervention. As noted above, clients who did not wish to participate were still provided with education about their diabetes using all components of the intervention but did not complete the pre- and postevaluation measures. Clients who agreed to participate were invited by the nurse to provide written consent. The generalist nurse then administered baseline questionnaires. Educational activities were undertaken as outlined above, according to each individual client's educational needs. Each client's involvement with the intervention varied from between one to three months depending on their educational requirements and length of episode of care with the service. Participating clients were then asked to complete the posteducation assessments. Data were collected before and after intervention using three scales of the HLQ [13] and the Diabetes Knowledge Questionnaire (DKQ) [14]. The DKQ is a 12-item multiple choice questionnaire that aims to measure knowledge change following a diabetes education intervention. There are two additional questions for people taking diabetes medication, and one for people with Type I diabetes. The questionnaire also asks for medication type and frequency, plus whether people have seen a diabetes educator or dietitian. The DKQ has been validated in Australian clinical settings [14].

All participating nurses were invited to take part in a postintervention semistructured interview to identify barriers to delivery of the intervention and any impact or changes in their clinical practice.

*Outcome Measures for Phase Three*. Outcome measures included changes to clients' knowledge about diabetes and changes in their ability to understand and use information about their diabetes. Evaluation consisted of completion of the DKQ and three scales of the HLQ prior to the intervention and completion of these same two questionnaires during an interview 8 weeks after the intervention. The three selected HLQ scales were as follows: (2) having sufficient information to manage my health; (5) appraisal of health information; and (9) understand health information enough to know what to do. Scale (5) was chosen as the comparison scale under the assumption that this aspect of health literacy was unlikely to be impacted upon by the intervention. We postulated that if there were no changes in the comparison scale, then this would suggest that any changes in the remaining two scales were more likely to be due to the intervention than not. The selection of scales (2) and (9) was based upon the program logic model, in which we identified that the intervention could be expected to impact on clients feeling they have sufficient information to manage their health and their ability to understand health information well enough to know what to do. A third scale identified by the program logic model, feeling understood and supported by healthcare providers, was not included to minimise respondent burden given that the Diabetes Knowledge Questionnaire and a comparison HLQ scale was also administered.

*Statistical Analysis for Phase Three*. Pre- and postintervention HLQ scale scores were analysed using effect sizes to estimate the change in scores. Interpretation of effect size was “small” >0.20–0.50, “medium” approximately 0.50–0.80, and “large” >0.80 [15]. DKQ scores were standardised to a possible score of 100 (as possible scores varied according to whether people were taking medication or whether they had Type I or Type 2 diabetes). DKQ were not normally distributed and are presented as medians and interquartile ranges (IQR). Data were analysed using STATA [11].

*Qualitative Analysis for Phase Three*. Interviews with nursing staff aimed to identify barriers to delivery of the intervention and any impact or changes in their clinical practice or for their clients. These data were analysed using NVivo Qualitative Software [16]. All transcripts were imported into NVivo in the initial stage. Themes were created deductively, guided by the stages of analysis as outlined by Colaizzi [17]. Any statement which was considered useful to the analysis was highlighted and coded as a node within NVivo. All transcripts were read in this manner, and the extracted significant statements were reread to gauge the embedded meanings. Thereafter a number of “mother” nodes reflecting these meanings were created, and related statements were grouped together and collapsed under the related mother node. A process of continual checking and rechecking between the transcripts and the nodes was undertaken to ensure the statements were being coded in the context they were spoken. The remaining transcripts were analysed and coded using the same process. NVivo's hierarchical tree structure for coding allowed the nodes to be classified, reclassified, and organised into main (mother) nodes and subnodes as required during this process.

### 2.3. Ethics

The study was approved by the Human Research Ethics Committees of the Royal District Nursing Service (project 138) and Deakin University (project 2012-295). Informed consent was obtained from all participants.

## 3. Results

### 3.1. Phase One: Health Literacy Assessment

One hundred and thirteen clients were recruited into the first phase of the study. The majority were female with a mean age of 75 years of age. The most commonly reported comorbidities were heart disease and arthritis (see Table 1).

Mean HLQ scores are shown in Table 2. Overall scores demonstrated that clients experienced difficulties in Scale (3), actively managing my health (mean 2.99, SD 0.42), and Scale (8), ability to find good health information (mean 3.55, SD 0.77). Many clients also reported difficulty with Scale (5), appraisal of health information (mean 2.78, SD 0.42). Higher HLQ scores were seen for Scale (1), feeling understood and supported by healthcare providers and Scale (6), ability to actively engage with healthcare providers (mean 3.23, SD 0.44 and 3.99 SD 0.57, resp.).

Cluster analysis produced thirteen clusters, each displaying a distinct pattern of responses to the HLQ. Cluster profiles ranged from lower to higher health literacy, and summary descriptors for each were developed, such as that who* has quite high health literacy but may be overwhelmed with information from too many sources*;* can understand health information when it is provided but is not active in health and feels unsupported by healthcare providers and others*; and* trusts healthcare providers but is not proactive or engaged with their own health*.

The workshop to develop potential intervention ideas was attended by six staff from the service including five Clinical Diabetes Educators and one Senior Research Fellow. During the workshop, a raft of factors that clinicians regarded as contributing to clients having such health literacy profiles were reported. Among the key issues identified were inconsistencies in the way diabetes education was delivered across the service, and the amount of information many clients accumulate (but do not necessarily engage with) from a range of sources. In total, 35 potential client-level and organisation-level responses to these needs were generated during the workshop, including educational focused strategies such as* not inundating patients with information; ensuring that education is provided in different ways; providing contextualised information; using teach-back to deliver information in small steps*; and* formal diabetes education for everyone.*

### 3.2. Phase Two: Codesign of the Intervention

**Figure 1:**
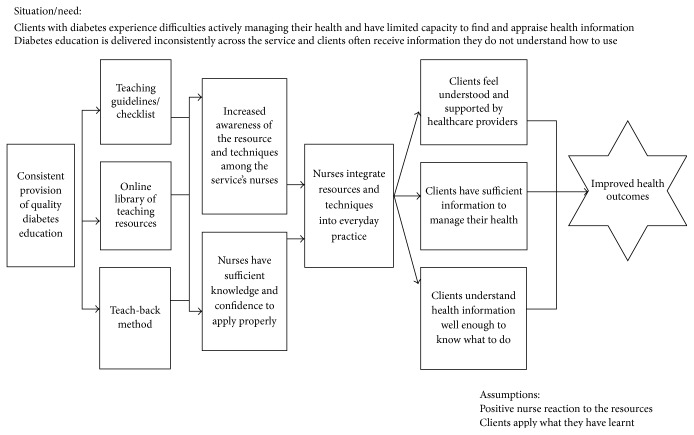
Program logic model for intervention.

**Figure 2:**
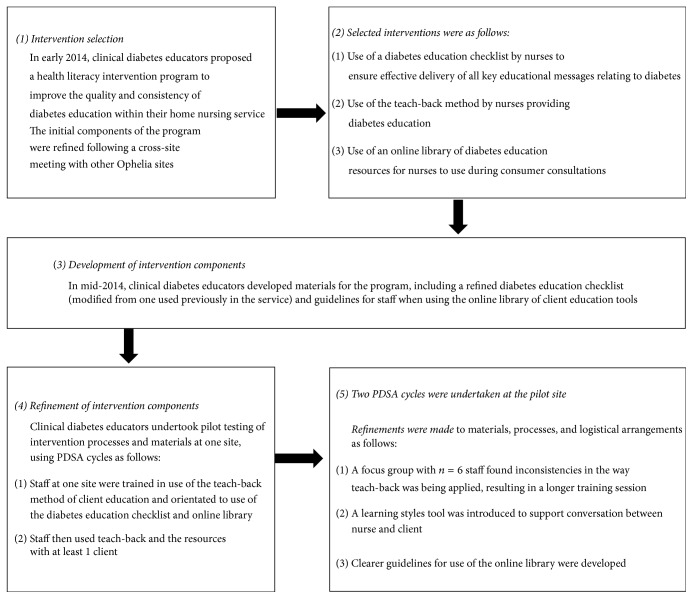
Intervention selection and development (Phase two).

The intervention ideas from the workshop were organised by the Clinical Diabetes Educators into a set of interventions suitable for use by generalist nurses that could be used to improve the quality and consistency of diabetes education within the nursing service provider. The researchers and Diabetes Educators then codeveloped a program logic model to identify how the intervention could achieve the project aims (Figure 1). The initial components of the program identified by the Clinical Diabetes Educators were then refined at a combined-site workshop in March 2014 (see Figure 2).

As shown in Figure 2, pilot testing of all processes and materials using PDSA cycles was conducted at one home nursing site where generalist nurses were trained in the use of the teach-back method of client education and orientated to use of the diabetes education checklist and online library resources. Nurses were asked to use the teach-back method with at least one client and to evaluate the checklist and resources. Two PDSA cycles were undertaken, with refinements made to materials, processes, and logistical arrangements as follows: (i) inconsistencies in the way teach-back was being applied led to longer training sessions, (ii) a learning styles assessment tool was introduced, and (iii) clearer guidelines for use of the online library were developed.

The final intervention consisted of three components:

(i) Guidelines and educational checklist are to be used by the home service nurses for education of clients with diabetes. Both resources were developed by the diabetes nurse specialist team.
(ii) Home nursing staff participating in the project were trained in use of the teach-back method [18]. This is a 4-step process in which clients are asked to repeat back information provided by the clinician in their own words to demonstrate understanding. Teach-back provided the opportunity for nurses to identify and clarify misunderstandings in relation to the client's ability to undertake diabetes self-management activities. The training session on teach-back provided nurses with the skills to adapt this method of education according to each client's personal context.
(iii) An online library of best-practice educational material was developed as a resource for nurses providing education to clients with diabetes.

Tailored diabetes self-management education was delivered in accordance with the client's preferred learning style. This was assessed using a learning styles assessment tool developed by another organisation participating in the larger Ophelia Victoria study and shared with RDNS to use as part of their intervention. The tool, which has not yet been validated, consists of a single page of pictures each depicting a method of learning. Clients were asked which of the methods they tended to use most when learning new information or tasks.

### 3.3. Phase Three: Intervention and Evaluation of the Final Intervention

**Figure 3:**
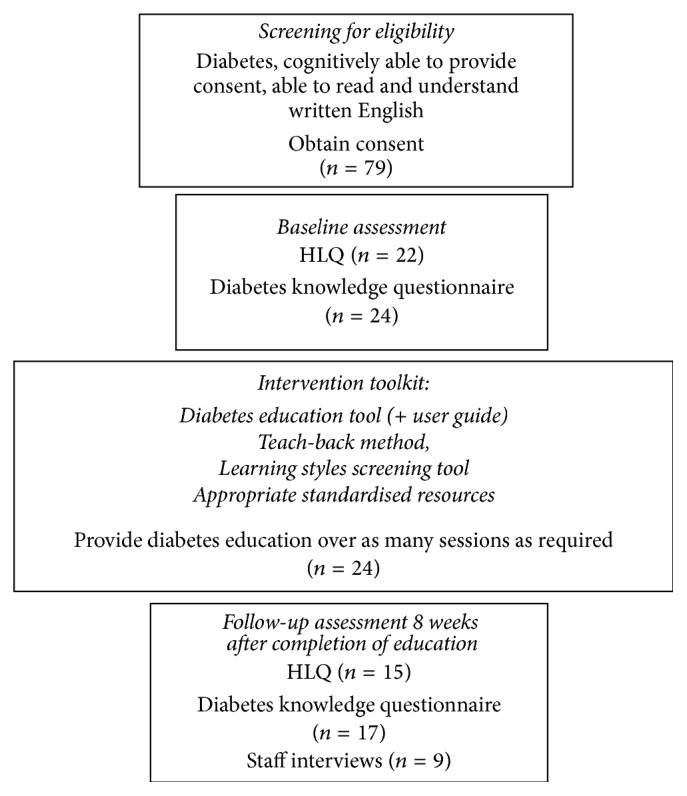
Flow diagram of Phase 3 of the Ophelia health literacy intervention showing client selection, intervention, and evaluation tasks.

A total of 79 clients were eligible to participate. Of these, 24 clients (16 females, 8 males) with a mean age of 75.3 ± 13 years (range, 51 to 98 years) agreed to participate in evaluation of the educational intervention (see Figure 3 and Table 3). While participants resided in a range of areas of according to the Australian Bureau of Statistics Socioeconomic Index for Areas (SEIFA) classifications [19] the majority (seventy-one percent) lived in areas categorised as disadvantaged.

**Figure 4:**
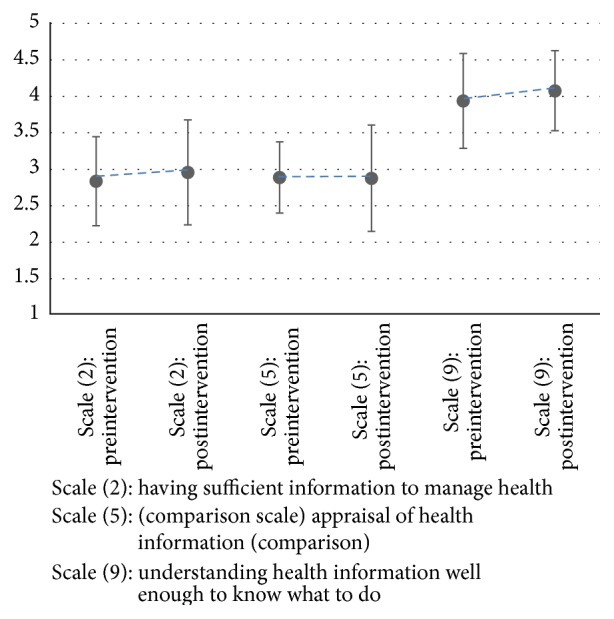
Changes in three HLQ scores before and after intervention (*n* = 15).

Twenty-two of the 24 clients recruited to the intervention study completed the pre-HLQ questions, with 15 of these completing both pre- and post-HLQ questions (Figure 4). As expected, no difference was seen in the comparison scale (Scale (5), appraisal of health information; mean prescore 2.93 (SD 0.51), postscore 2.91 (0.74). Effect size (ES) 0.04, 95% CI −0.67, 0.76). Minimal positive increases were seen in the remaining two scales (Scale (2), having sufficient information to manage health; mean prescore = 2.88 (0.59), postscore = 2.98 (0.72). ES = 0.15, 95% CI −0.57, 0.87), and (Scale (9), understanding health information well enough to know what to do; mean prescore = 4.04 (0.49), postscore = 4.08 (0.57). ES = 0.08, 95% CI −0.64, 0.79).

All 24 clients completed the preintervention Diabetes Knowledge Questionnaire and 17 completed both pre- and postintervention (see Table 4). Trends suggested an overall increase in median DKQ scores but this difference was not statistically significant.

### 3.4. Nurses' Perceptions of Barriers to Implementation and the Utility of the Intervention and Its Impact on Their Clinical Practice and Client Outcomes

Twenty-four nurses attended training sessions of which 13 recruited clients and delivered education. Nine of the 13 nurses participated in interviews to report on their perceptions in relation to barriers and utility of the intervention and any impacts for themselves or their clients.

A total of six themes were identified in the NVivo analysis. There were five strong themes and a sixth weaker theme relevant to the use of the learning styles tool (see Table 5). Strong themes encompassed those where a minimum of five participant responses supported key trends, while the weaker theme involved responses by only two participants.

## 4. Discussion

This study describes a systematic process that enabled a home nursing service to identify and respond to the health literacy strengths and challenges of their clients with diabetes. The Ophelia process allowed the service to lead the collection of health literacy data, participate in codesign workshops, codevelop their own program logic models, apply quality improvement cycles, and then lead the implementation and evaluation of an effective intervention. In this setting, the Ophelia process is shown to be a feasible approach by which an organisation can understand and respond to the health literacy needs of their clients and build health literacy capacity of staff and the organisation itself.

Overall findings suggested small improvements in outcomes. There were slight, but not significant, increases in the two HLQ scale scores used for evaluation and in the Diabetes Knowledge Questionnaire scores. In addition, the generalist nurses indicated positive behaviour changes for some clients and a greater rapport between nurse and client. There were also clear benefits for generalist nursing staff to using a consistent approach and expected standard for diabetes education delivery, with a dedicated resource hub and the diabetes education checklist now embedded into usual practice for assessment of client education needs. The “teach-back” education method has been identified as a skill for staff development and has been advocated for use across the home nursing service training/education programs as part of the effort to educate generalist nurses on health literacy and practical intervention and support. The intervention is thus becoming part of routine clinical practice and will become embedded within the organisation over time. Due to a new remote working environment of the home nursing service, teach-back training may need to be delivered using online learning modules, supported by regional Clinical Diabetes Educators. In addition, the Diabetes Clinical Educators and Senior Clinical Nurse Advisor for dementia will collaborate to ensure the intervention is suitable for the needs of clients with dementia. In this way, the intervention is tailored to meet the changing needs of the organisation and its clients.

The intervention was derived from a detailed needs assessment of the client group, and the use of teach-back and assessment of learning styles allowed further tailoring of education to client's individual needs. Similar findings were seen in a US community clinic, where the use of educational materials targeted to health literacy levels and learning styles was found to increase clients' diabetes knowledge compared to those not receiving the tailored intervention [20]. A systematic review of the efficacy of tailored interventions for self-management in chronic disease found that among clients with diabetes, the provision of tailored information was associated with improved self-care behaviours and knowledge [21]. The author also found that development of a personal rapport or relationship with the person providing the information was an important component of the intervention [21]. Personal rapport and empathy have been shown to be related to health outcomes [22] and may have been a contributing factor to the success of our intervention in which education was generally provided by the same nurse over a period of time, allowing for a positive relationship to develop. Similarly, a systematic review of the effectiveness of diabetes self-care interventions found that healthcare provider support and health literacy influenced people's self-care ability, with findings from this review also suggesting that using approaches that are tailored to the needs of different groups of people with diabetes are effective [23].

Other studies examining the effectiveness of teach-back for clients with diabetes have shown similar findings, even where patients have lower health literacy. A frequently reported study from North America found that physicians' use of teach-back was associated with improved glycaemic control among patients with diabetes mellitus and low functional health literacy [24]. A study from Iran found that among patients with type 2 diabetes and low health literacy attending a diabetes outpatient clinic use of teach-back was associated with improved knowledge about diabetes and improved adherence to medication, maintained at 6 weeks after intervention [25]. Use of teach-back was also associated with knowledge recall among community-based patients with type-2 diabetes in the USA; however, knowledge retention was not maintained at 2 weeks [26].

Involvement of the nurse Clinical Diabetes Educators in all stages of the process (from data collection to evaluation of the intervention) ensured ownership of the intervention and empowered the Diabetes Educators. It also meant that the Educators were able to support the service's generalist nurses to understand and apply the intervention, by explaining the project in words that their colleagues understand, and using practical and relevant examples. Further, the close involvement of the Educators meant that the organisational context, structure, and culture were considered when designing the intervention. Understanding the context of a person's daily life and knowledge of the healthcare service is an important factor in the design of health literacy interventions. Health literacy is very context-specific [7], and so interventions delivered in one context or to one group of patients may not be as effective in another, even if people have similar health literacy abilities. The Ophelia process applies a codesign approach that takes into account the knowledge of clinicians who are not only very experienced clinically but who have also worked with the client target group for some time and so are familiar with many of their day to day health literacy challenges and abilities.

### 4.1. Study Strengths and Limitations

This is the first time this process has been used in a large home nursing service and was a proof-of-concept study with limited outcome data; however, our findings demonstrate that a health literacy intervention can be generated and applied in this setting using the Ophelia approach.

A major restructure of the home nursing service occurred during this project, including the introduction of remote service delivery. These changes led to a delayed start to Phase three of the project, impacting upon the numbers of study participants recruited and reducing the available time for intervention implementation and evaluation. Further, participants who completed both the pre- and posthealth literacy and diabetes knowledge questionnaires are likely to be those who have greater self-management skills and possibly higher health literacy and therefore results are not likely representative of the wider client population. Many people with low health literacy are not likely to have taken part and therefore there are limits to the transferability of the results to this group in particular. Strategies for ensuring that clients who are appropriate for engaging with the interventions, that is, including those with a range of substantial health literacy challenges, will need to be explored further and a stratified approach used for those who are unable to engage with the planned intervention to ensure maximal independence and safety is maintained. In addition, the learning styles assessment tool was not validated prior to its use and so cannot be said to accurately assess preferred learning styles. In order to provide a strong evidence base we recommend that our model requires further testing and a wider scale evaluation.

## 5. Conclusion

The organisation will continue to evaluate and develop a consistent and deliverable diabetes education program that responds to the needs of a diverse client population with varying health literacy strengths and limitations. From participating in this process, staff and management now have a greater understanding of the relevance of health literacy for their clients and increased knowledge of how to develop interventions based on these needs. In this setting, the Ophelia process has contributed to evidence-informed practices changes that, to date, have been maintained.

## Supplementary Material

The Diabetes Education Tool and accompanying User Guide used in this intervention can be found in the Supplementary Material.

## Figures and Tables

**Table 1:** Demographic and health profile of participants who completed initial health literacy needs assessment (*n* = 113).

Variable name	*n* (%)
Age (mean, standard deviation)	75 ± 10.0
Female	61 (55%)
Lives alone	58 (53.2%)
Australian born	73 (65.2%)
Main language	103 (92.0%)
Part secondary education or less	78 (69.7%)
Private health insurance	37 (33.9%)
Healthcare card	99 (88.4%)
Assisted with questionnaire	73 (65.7%)
Arthritis	55 (49.6%)
Back pain	41 (36.6%)
Heart problems	60 (53.6%)
Respiratory	16 (14.3%)
Cancer	15 (13.4%)
Depression and/or anxiety	35 (31.3%)
Diabetes	107 (95.5%)
Stroke	17 (15.2%)
Other conditions	34 (30.1%)
Reported no health condition	1 (0.3%)

**Table 2:** Health literacy questionnaire (HLQ) scale scores.

HLQ scale		Mean (standard deviation)
Possible scores for these scales range between 1 & 4	(1) Feeling understood and supported by healthcare providers	3.23 (0.44)
	(2) Having sufficient information to manage my health	3.02 (0.43)
	(3) Actively managing my health	2.99 (0.42)
	(4) Social support for health	3.07 (0.48)
	(5) Appraisal of health information	2.78 (0.42)

Possible scores for these scales range between 1 & 5	(6) Ability to actively engage with healthcare providers	3.99 (0.57)
	(7) Navigating the healthcare system	3.79 (0.60)
	(8) Ability to find good health information	3.55 (0.77)
	(9) Understanding health information well enough to know what to do	3.72 (0.72)

*For scales (1) to (5):* a score of 1: strongly disagree; 2: disagree; 3: agree; 4: strongly agree.

*For scales (6) to (9):* a score of 1: cannot do or always difficult; 2: usually difficult; 3: sometimes difficult; 4: usually easy; 5: always easy.

**Table 3:** Intervention participant demographics.

Age	Mean (SD) = 75.3 ± 13.2, range 51 to 98
Gender	Female: *n* = 16 (66.7%); male: *n* = 8 (33.3%)
Years with diabetes	Mean (SD) = 9.78 ± 9.5, range 0.1 to 35 *n* = 23 of 24 clients had type 2 diabetes (*n* = 1 missing data)
Medication type	Oral medication only (*n* = 9, 37.5%); insulin only (*n* = 5, 20.8%); both (*n* = 9, 33%); none (*n* = 1, 4%); missing (*n* = 1, 4%)
Ever seen diabetes educator	Yes = 18 (75%); no = 6 (25%)
Ever seen dietitian	Yes = 14 (58.3%); no = 10 (41.7%)
SEIFA index of relative disadvantage^*∗*^	SEIFA < 1000, *n* = 17 (71%)
	SEIFA ≥ 1000, *n* = 7 (29%)

^*∗*^ABS: socioeconomic indexes for areas (SEIFA) index of relative disadvantage [19]. Note: a lower score indicates that an area is relatively disadvantaged compared to an area with a higher score. Index scores have been standardised to have a mean of 1,000.

**Table 4:** Changes in diabetes knowledge questionnaire score (DKQ).

	Median (interquartile range)
*Participants only completing preintervention DKQ (n* = 24)	
Pre-DKQ score	75 (62, 89)

*Participants completing pre- & postintervention DKQ (n* = 17)	
Pre-DKQ score	77 (65, 88)^*∗*^
Post-DKQ score	89 (77, 96)

Possible score range for the DKQ = 0 to 100.

^*∗*^No significant difference between median scores using Wilcoxon signed rank sum test.

**Table 5:** Key themes and illustrative quotes.

Themes	Findings	Illustrative quotes
Benefits experienced during the use of diabetes education checklist	Six nurses reported that the checklist helped them keep on track with client education by focusing only on areas the client thought were necessary. Overall, the checklist appeared to be well accepted and utilised and was termed “user-friendly”	I think it was useful – in her situation I was the only one giving her the education, when lots of different nurses – where it's good to have different ideas you sometimes end up guessing what has been covered, often re-hashing and going over time that has already been spent, making sure that you haven't missed, whereas if doing all education,…in that conversation you realise that oh they didn't know that, useful conversation around what do you know/ not know. (RDNS 7) I tend to use the checklist now for all my diabetes clients - this is much easier for me to tick off what they need to learn (RDNS 4)

Benefits and barriers experienced during the use of teach-back	The method was praised by most nurses (*n* = 7) who felt that while it had been part of their routine clinical practice for some time, participating in this intervention led to consistent and conscious use of the method during client education. Using the method more formally was seen to reinforce the importance of the teaching and learning trajectory to both clinicians and clients. The nurses (*n* = 7) reported that using teach-back raised their awareness of the needs of clients in relation to learning such as the need to provide information in stages, use of simple terms, and being specific about actions that clients needed to undertake. The method was seen as contributing to a greater rapport with clients (*n* = 4). Using the method with dementia patients and other cognitively impaired patients was a challenge identified by two clinicians	I felt confident straight away to practice – was already using techniques, but the project made me more aware and made me use it more consciously and consistently (RDNS 1) I spend a lot more time asking patients what was the main thing they understood from that and encouraging them to talk back to me. Before I was more “you've heard the information now go and do it”. It was reinforcing to me about my teaching, she and I both enjoyed it (RDNS 2) With teach-back, I think it's a great way to communicate with people – we say “this is what we are going to do”, not “this is what you need to do”. We work with them and get a better response all round. (RDNS 5)

Benefits and barriers experienced during the use of online library of resources	Five clinicians noted that these resources were “useful for quick answers,” “user-friendly,” and “easy to use.” However, two clinicians felt the topics were limited, and sharing the resources with clients was challenging when large/multiple documents needed to be downloaded, printed, and mailed out to clients	I use the diabetes education checklist and online resources all the time with other clients. They are good, they help keep me on track and remember what I've covered (RDNS 5) I used all online resources – they are written in simple language, a couple I got from the National Diabetes site, plus shopping list off the diabetes website – a very useful tool (has product names on it, much more practical) (RDNS 2) Then there is still same problem with accessing resources – large documents that have to be downloaded – we need to print them as that's the only way I can give to people to read – not enough time in our meeting to read over again in our session, screens too small, especially if lots of sections – do people really bother to read them all? (RDNS 7)

Benefits and barriers experienced during the use of the learning styles tool	Only two nurses specifically reported using this tool; one nurse felt it made educating staff easier and was a user-friendly tool to use, while the second reported that using the tool with older clients, who had set habits, was a challenge	I used the learning styles tool initially, thought that was useful but I do that anyway (RDNS 2) The learning styles – I think that's important, but with our kind of clients, we don't really have the ability to do things differently. We'll go in and talk about things – if they need resources we'll do what we can. With the age of our clients, what they're used to is us sitting down with them – it's not practical to know about their learning styles (RDNS 5)

*Experiences and outcomes*	Two strong subthemes here were the “opportunities” and “challenges” which arose during utilisation of the intervention's tools. There were positive reports by three nurses of clients becoming more proactive, asking more questions and showing improvements in self-management of their condition. Nurses (*n* = 5) felt this was a result of increased knowledge, understanding, and opportunity for clients to refresh their memory on certain vital topics. In terms of changes to their own knowledge and practice, two nurses reported no changes, while five reported that the intervention provided opportunity to reflect on how education was delivered to clients and taught them to look for cues to ascertain client understanding of the content. Nurses (*n* = 5) felt the intervention either formalised the process of information delivery, and/or provided an opportunity to check on a client's existing knowledge, refresh knowledge, and build rapport with a client. In relation to challenges, five nurses reported that recruiting suitable clients to participate in the intervention was difficult given the large proportion from non-English speaking backgrounds. The second biggest challenge was client cognitive decline or impairment as noted by two nurses	She's more confident to ask questions. She has had a foot wound which she has stopped looking after, so she has asked me if anything else, and I said well let's do foot care, so we've done more about this and got her to a podiatrist, so definitely more proactive than previously. I've known her for 3 years, and this is different. (RDNS 2) Has given me an opportunity to reflect on how I deliver education and reflect back and look at what I've done more analytically and see that nodding the head doesn't mean they get it…looking for objective and subjective cues about how they have learnt (RDNS 6) I found only one suitable person, because limited criteria I have many patients with non-English speaking background or cognitive decline (RDNS 3)

*Critical facts and lessons learnt*	Cultural and linguistic diversity was predominant amongst the target population and therefore translation and use of simple language were suggested to make the intervention more relevant (*n* = 5). The continuous promotion of the intervention within the organisation was advised to maintain its momentum. In terms of client behaviours, staff (*n* = 3) felt some clients/carers may dislike being assessed/questioned on topics they had limited knowledge about. Allowing clients to learn at their own pace, educating them without impeding their confidence, and encouraging clients to be independent were suggested as vital points by clinicians (*n* = 3). One nurse reported that using the tools with some clients revealed cognitive issues which had not been previously identified, due to a lack of formal assessment. Finally, nurses praised the Clinical Diabetes Educators who led the project from within the home nursing service for their supportiveness, availability, and responsiveness	CNCs will need to keep promoting it. If there is no one driving it, it won't be successful (RDNS 1) If we are going to take education seriously, we should use this method- each site in RDNS is doing something different. Not to say it's bad but to be consistent, we need consistent methods…incorporating teach back is the first tool (RDNS 6) We don't encourage our patients to be independent (RDNS 3) Think it is a good idea, but can see that many people would benefit from education, but not all are English speaking, so some translation required (RDNS 3) It comes with practice and being aware that everyone is at different stages, some will take longer, and need to go over and over, some people take it in quickly. Need to be really patient with people (RDNS 8)
