# Supplementary material for: Optimising Health Literacy and Access of Service Provision to Community Dwelling Older People with Diabetes Receiving Home Nursing Support
Source: J Diabetes Res. 2016 Sep 7;2016:2483263. doi: 10.1155/2016/2483263 (PMC5030436; doi:10.1155/2016/2483263)
Supplement: Supplementary file 1 — The Diabetes Education Tool and accompanying User Guide used in this intervention can be found in the Supplementary Material. [file 2483263.f1.pdf]

Diabetes Education Tool

Attach client label if available
Name:
UR:

This form is used in conjunction with the resources available via the CLG Diabetes webpage/Diabetes Resource Hub.

	Date discussed	Resource provided	Not applicable	Comments
**DIABETES –** discuss type specific to referral				
What is diabetes?			☐	
**BLOOD GLUCOSE MONITORING**				
When and why to test			☐	
Equipment			☐	
Technique for testing			☐	
Recording and interpreting results			☐	
Blood ketone testing			☐	
**DIABETES MEDICINES**				
Medicine name, dosage, frequency and action			☐	
Injectable devices(insulin & GLP-1's)			☐	
Storage of injectables			☐	
Injectable technique			☐	
Sharps disposal/where to obtain			☐	
NDSS (National Diabetes Services Scheme)			☐	

	Date discussed	Resource provided	Not applicable	Comments
**HEALTHY EATING**				
Healthy eating principles			☐	
**KEEPING ACTIVE**				
Regular activity			☐	
**HYPOGLYCAEMIA**				
Signs/symptoms			☐	
Causes			☐	
Treatment			☐	
**HYPERGLYCAEMIA**				
Signs/symptoms			☐	
Causes			☐	
Treatment			☐	
**SICK DAYS**				
Plan				
**REGULAR CHECKS**				
Foot care			☐	
Eye care			☐	
Dental care			☐	
Kidney care			☐	
**LIFESTYLE CONSIDERATIONS**			☐	
Smoking			☐	
Alcohol			☐	
Driving			☐	
Travel			☐	
**OTHER:**			☐	

	Date discussed	Resource provided	Not applicable	Comments
			☐	
			☐	
**OTHER COMMENTS:**			☐	
			☐	
			☐	
			☐	
			☐	

Diabetes Education Tool User Guide

The *Diabetes Education Tool* is used by nurses to record the diabetes education needs of clients and as a checklist of the topics covered and resources supplied.

The *Diabetes Education Tool* is to be used in line with RDNS policies and procedures.

ITEM	DEFINITION/DESCRIPTION
**GENERAL INFORMATION**	Diabetes education is a major component of diabetes management. This guide for the "Diabetes Education Tool" is to be used in conjunction with the diabetes nursing care plan and the diabetes education resources available on the RDNS CLG Diabetes Resource Hub.<br><br>When providing diabetes education consideration must be given to each individual client's learning style as well as their cultural and educational background. This tool is designed for use as a check list to guide the education delivered to the client. Education is individualised and only sections relevant to the client need to be completed. The educational resources available on Pulse used in conjunction with the Diabetes Education Tool and User Guide provide basic diabetes information. If your client requires more complex information or assessment please refer to a CNC Diabetes RDNS or a Diabetes Educator in the community.
**Date discussed**	Write the date on which you discussed the topic with the client/carer.
**Resource provided**	If any resources (eg. brochures, handouts) have been given to the client/carer, specify what they are.
**Comments**	Include any other relevant information
**DIABETES**<br><br>**What is diabetes?**	• Provide a simple explanation. You may like to use the term sugar or glucose depending on client's comprehension.<br>• Explain both Type 1 and Type 2 Diabetes so the client has a better understanding of the differences.<br>• Elaborate on the client's specific type of diabetes.
**BLOOD GLUCOSE MONITORING**<br><br>**1. When and why?**	• Monitoring is individualised to client need.<br>• Good times to check are prior to main meals or 2 hours post a meal, or more frequently in times of illness.<br>• RDNS policy recommends BGL's be checked before insulin administration.<br>• BGL monitoring assists health professionals (e.g. treating Doctor) to provide advice on diet, activity and medication management and empowers clients to make improved choices.
**2. Equipment**	• If the BGL meter is greater than 5 years old consider replacement. Most BGL meter companies will upgrade old or damaged meters free of charge and will also provide a free battery annually. Modern BGL meters require less blood, therefore are more user friendly for the client.<br>• Strips need to be stored in their original packaging. Keep away from moisture, direct sunlight and heat. Check expiry and also discard used strips,

	or strips with damaged packaging or open lids. All BGL meters less than 2 years old no longer require calibration with each new packet of strips.<br>• A lancet pen device (e.g. softclix or multiclix) should only be used by clients who are independent with care. If the client is not able to obtain their own BGL, or change their lancet pen needle/barrel then a disposable single use lancet device (e.g. Unistix3) should be implemented and the lancet pen device should be removed from RDNS work space.<br>• Discuss with the client the frequency of lancet pen needle changes and recommend regular changes dependent on how frequently the client attends their BGL.
**3. Technique for Testing**	• Wash hands with warm soapy water and dry thoroughly. Alcohol wipes or hand gels are not recommended.<br>• Encourage rotation of fingers used (thumb included) to obtain blood sample.<br>• Observe the client attend BGL to ensure done with correct technique.
**4. Recording and Interpreting Results**	• Discuss client's individualised BGL target levels, this may need to be done in consultation with their treating doctor.<br>• Encourage clients to record BGL levels in a log book or diary.<br>• BGL meters store a memory of results, time and date should be set correctly.
**5. Blood Ketone Testing**	• People with Type 1 diabetes should be aware of blood ketone testing to prevent progression to DKA.<br>• Blood ketone testing can only be performed on a Free Style Optium meter with blood ketone strips. This monitor prompts to check ketones (displays 'KETONES?') on the screen if BGL is greater than 15mmol/L.<br>• Blood ketone testing information is available on RDNS Diabetes CLG resource hub within resource folder.
**DIABETES MEDICINES**<br><br>**1. Medicine name, dosage, frequency & action**	• Refer to MIMS or Australian Medicines Handbook (available on Pulse)<br>• Refer to resource folder on the RDNS Diabetes CLG hub under Resource folder for insulin profiles
**2. Injectable devices**	• Refer to the RDNS Diabetes CLG resource hub under Resource Folder for information regarding different devices.
**3. Storage of injectables**	• All injectables not in use should be stored towards the bottom of the fridge. In use devices can be stored out of the fridge for 28 days away from direct sunlight, heat and cold.
**4. Injectable technique**	• Observe client technique. If the client is using a pen device ensure they are aware to attend a 2 unit air shot. This will prime the needle and also confirms the needle is functioning.<br>• Recommend the needle/syringe be changed each injection<br>• Once dose has been administered ensure the client keeps the needle in-situ for 5-10 seconds prior to removing from the skin.
**5. Site Rotation**	• The abdomen is the preferred site of administration for s/c insulin as absorption rate is predictable.<br>• Recommend site rotation to prevent lipo-hypertrophy or bruising. Injection

	into the same site on multiple occasions can alter absorption of insulin.<br>• The entire abdomen can be used. Avoid 2cm's around umbilicus.
**6. Sharps disposal/where to obtain**	• Lancet needles, single use lancets (e.g. unistix3), injectable pen needles and syringes must be disposed directly into a regulated sharps bin.<br>• Injectable pens with no needle attached can be disposed into general waste.<br>• Sharps bins are available free of charge from the local council or can be purchased from pharmacies.
**7. NDSS (National Diabetes Services Scheme)**	• NDSS is a free service administered by Diabetes Australia providing some subsidised diabetes equipment.<br>• Registration forms via NDSS website.
**HEALTHY EATING**	
**Healthy Eating principles**	• Refer to RDNS CLG Diabetes Resource Hub for information.<br>• Refer to Dietician for more specific dietary recommendations/advice.
**KEEPING ACTIVE**<br><br>**Regular activity**	 <br>• Set realistic goals for individualised care.
**HYPOGLYCAEMIA (HYPO)**	
**1. Signs /Symptoms**	• Signs and symptoms may not always be present, this is referred to as 'hypo unawareness' and extra precaution should be taken, such as more frequent BGL monitoring.
**2. Causes**	• Discuss possible causes such as an increase in physical activity, skipping of meals and loss of weight. Discuss how to prevent hypoglycaemia in the future. Consider liaising with the treating Doctor as medications may need to be reviewed and reduced.
**3. Treatment**	• Clients or their carer must be aware of actions to take in the event of a hypo. Clients should always have available fast acting glucose in the home or on them at all times.
**HYPERGLYCAEMIA**	
**1. Signs /Symptoms**	• There are many signs and symptoms that go unnoticed; however sustained hyperglycaemia is detrimental to the health and wellbeing of an individual.<br>• Discuss Diabetic Ketoacidosis (DKA) or Hyper Osmolar Non-Ketotic (HONK) if applicable.
**2. Causes**	• Concurrent illness, medicines, stress and diet, are some of the possible causes of hyperglycaemia.
**3. Treatment**	• Sometimes high BGL's can be a one-off (e.g. dietary intake). Problem solving with the client to find the reason behind hyperglycaemia is essential however can be quite difficult and immediate action cannot always be taken. It is important that regular BGL monitoring is attended and reporting to the treating Doctor is paramount if cause cannot be determined.
**SICK DAYS**	

**Plan**	• Be prepared, plan ahead. Consider providing the sick day management plan section from the RDNS Plain English Information sheets.
**REGULAR CHECKS**	
**1. Foot Care**	• Consider diabetes foot education utilising the Foot Ulcer Client Education Tool available for download on Pulse<br><br>• Consider referral to Podiatrist via the Enhanced Primary Care Scheme (EPCS), completed by GP.
**2. Eye Care**	• Eye checks are free via Medicare at any Optometrist. Encourage the client to notify the Optometrist they have diabetes.
**3. Dental**	• Even if the client has dentures they still need regular dental reviews.
**4. Kidney Care**	• Regular reviews with GP or specialist is essential. Encourage the client to discuss this with their treating Doctor. Renal function reviews may include blood tests, urine tests and BP monitoring.
**LIFESTYLE CONSIDERATIONS**	
**1. Smoking**	• Consider referral to QUIT program
**2. Alcohol**	• Alcohol can influence diabetes medicine actions which can lead to hypoglycaemia. Encourage carbohydrate consumption when alcohol is consumed.
**3. Driving**	• It is the client's responsibility to notify their local driving authority (e.g. Vic Roads) of their diabetes.<br>• Fitness to drive should be discussed with the treating Doctor.<br><br>**References**<br><br>• Diabetes Australia Victoria 2008, "Health Professionals, Diabetes resources, Diabetes Information sheets" 'avail at:' http:// www.diabetesvic.org.au<br>• Rdns 2011,"Diabetes information sheets in plain English" 'avail at:" http// www rdns.com.au.<br>• ADEA 2011,"**Clinical Recommendations – Subcutaneous Injection Technique for Insulin and Glucagon-like Peptide1 i**nsulin guidelines," 'avail at,' http// www.adea.com.au.
